# Influence of Length-to-Diameter Ratio on Static and Dynamic Behavior of Rocks: A Review and Perspective

**DOI:** 10.3390/ma18051109

**Published:** 2025-02-28

**Authors:** Kang Peng, Yansong Bai, Song Luo, Kun Luo, Haoyu He, Ze Xi

**Affiliations:** 1 School of Resources and Safety Engineering, Central South University, Changsha 410083, China; pengkang2020@csu.edu.cn (K.P.); 225511017@csu.edu.cn (Y.B.); 245511013@csu.edu.cn (H.H.); 235511033@csu.edu.cn (Z.X.); 2 School of Resources Environment and Safety Engineering, University of South China, Hengyang 421001, China

**Keywords:** rock mechanics, length-to-diameter ratio effect, static mechanics, dynamic mechanics, failure mode

## Abstract

The size effect in rock mechanics has long been a challenging issue, with the length-to-diameter (L/D) ratio emerging as a critical factor that has received substantial attention. Understanding the L/D ratio effect is essential for ensuring the accuracy and reliability of laboratory tests. This paper presents a comprehensive review of studies on the L/D ratio effect in rocks, examining its influence on static and dynamic mechanical behavior. The analysis encompasses the effects of specimen L/D ratio on rock mechanical properties, energy characteristics, and failure modes, integrating the findings from theoretical research and experimental studies. The results reveal that the L/D ratio effect varies significantly under different external loading conditions. Despite extensive research, the mechanisms underlying the L/D ratio effect remain ambiguous, and a unified conclusion has yet to be reached. This review highlights the importance of selecting appropriate specimen dimensions for laboratory tests and emphasizes the need for further investigation into the L/D ratio effect to advance the understanding of rock mechanics.

## 1. Introduction

In rock mechanics, the size effect is a fundamental and widely studied phenomenon, referring to the significant variation in mechanical properties of rock samples as their size changes. This effect is crucial for understanding the mechanical behavior of rocks at different scales and evaluating their performance in engineering applications. The concept of the size effect was first introduced at the First International Symposium on Rock Mechanics in 1950, and it has since garnered considerable attention [1]. Despite ongoing research, it remains an unresolved challenge in rock mechanics. Zhao et al. [2] describe it as one of the “century-old problems” that continues to elude resolution in the field.

The size effect manifests in various forms, with the L/D ratio (the ratio of a rock specimen’s length to its diameter) being among the most influential parameters [3,4,5]. As a critical geometric characteristic, the L/D ratio determines the stress distribution within the rock during loading, directly affecting its failure mode and strength characteristics [6,7,8]. Variations in the L/D ratio significantly influence stress concentration, crack propagation paths, and the overall strength of rocks, with these effects differing under static and dynamic loading conditions. Therefore, an in-depth investigation of the impact of the L/D ratio on the mechanical properties of rocks is crucial for understanding the mechanical responses of rock masses under various engineering conditions.

In recent years, research on the influence of the L/D ratio on the mechanical properties of rocks has advanced significantly. Under static conditions, the L/D ratio significantly affects the stress-strain behavior, failure modes, and strength characteristics of rocks in compression and tensile tests [9,10,11,12,13,14]. In dynamic mechanics research, the split Hopkinson pressure bar (SHPB) is commonly used to investigate the effects of the L/D ratio on the dynamic strength, failure modes, and energy evolution characteristics of rocks under high strain rates [7,15,16,17]. It should be noted that although the size effect of rocks is widely discussed in rock mechanics research, the core focus of this paper is the L/D ratio effect. The L/D ratio primarily examines the influence of the ratio of the length to the diameter of a rock specimen on its mechanical properties [18], particularly with respect to strength and failure modes under both static and dynamic loading conditions [7]. In contrast, the sample volume effect focuses more on how variations in the rock specimen’s volume impact its mechanical behavior. While there may be some overlap between these two effects in certain studies [19,20,21], this paper will concentrate on the L/D ratio effect, avoiding a deviation from the main topic by introducing discussions on the volume effect.

From the perspective of rock mechanics experiments and engineering applications, understanding the impact of the L/D ratio is crucial for experimental design and the safety assessment of practical engineering projects. The appropriate selection of the L/D ratio not only ensures the accuracy of experimental results but also provides reliable guidance for rock mass stability analysis, tunnel construction, and deep foundation engineering. In geotechnical engineering projects, engineers often design based on the mechanical properties of rocks, and the choice of L/D ratio directly affects the reliability of experimental data and the accuracy of rock mass mechanical parameters. Therefore, the research presented in this paper provides a theoretical foundation for rock mechanics experiment design and offers valuable insights for safety assessments and design optimization in geotechnical engineering projects.

This paper systematically and comprehensively analyzes the impact of the L/D ratio on the mechanical properties of rocks from both static and dynamic perspectives, and it discusses in detail the influence of the L/D ratio on rock strength, failure modes, and energy evolution patterns, based on experimental data analysis. Under static loading conditions, the impact of L/D ratios on uniaxial compression, triaxial compression, and tensile tests is reviewed. Under dynamic loading conditions, the effects of L/D ratios on dynamic compression and tension are outlined. This research provides a robust theoretical foundation and practical reference for the design and safety evaluation of rock mass engineering projects.

## 2. Influence of Length-to-Diameter Ratio on Static Mechanical Properties of Rocks

The selection of the L/D ratio for rock specimens plays a crucial role in determining their static mechanical properties. Systematic experiments and analyses provide insights into how the L/D ratio affects rock strength, failure mode evolution, and energy dissipation. This chapter will explore, in detail, the effects of the L/D ratio on the mechanical properties observed in uniaxial compression tests, triaxial compression tests, and tensile tests.

### 2.1. The Length-to-Diameter Ratio Effect in Uniaxial Compression Tests

Uniaxial compressive strength (UCS) is a key mechanical parameter widely used to characterize the properties of rocks. In uniaxial compression tests, the L/D ratio has a significant effect on the UCS of rocks. Studies have demonstrated that UCS varies with the L/D ratio [4]. Table 1 presents the compressive strength data of 20 rock samples tested at two different L/D ratios, as reported in the literature. For specimens with an L/D ratio of 1, the UCS is higher than that of specimens with a ratio of 2. When the UCS of specimens with L/D = 1.0 is converted to that of standard specimens (L/D = 2.0), the conversion factor ranges from 0.76 to 0.98 [10]. This conversion factor reflects the differences in UCS between specimens with varying L/D ratios. A lower conversion factor corresponds to a greater difference in UCS between specimens.

To standardize the UCS of specimens with L/D ratios smaller than 2.0, researchers have proposed two conversion formulas [22,23]. These formulas are as follows:

(1) $$UCS_{P}=UCS_{L}/(0.88+0.24D/L)$$

(2) $$UCS_{P}=8UCS_{L}/(7+2D/L)$$

where *UCS_P_* is the predicted UCS for the standard specimen (L/D = 2.0), and *UCS_L_* is the UCS obtained from experimental tests on specimens with an L/D ratio less than 2.0.

**Table 1:** Compressive strength and conversion factors of rocks at different L/D ratios (*UCS*_1_ refers to the UCS at L/D = 1; *UCS*_2_ refers to the UCS at L/D = 2).

Rock Type	*UCS*_1_ (MPa)	*UCS*_2_ (MPa)	Conversion Coefficient	Data Sources
Sandstone	101.32	82.53	0.81	Hawkins [24] (1998)
Marble	72.97	63.14	0.87	Zhu et al. [25] (2004)
Limestone	120.75	106.32	0.88	
Marble	59.90	50.63	0.85	Yang et al. [26] (2005)
Rock salt	29.50	26.00	0.88	Özkan et al. [27] (2009)
Basalt	67.30	61.30	0.91	Tuncay and Hasancebi [4] (2009)
Gray andesite	129.40	102.10	0.79	
Pink andesite	74.90	70.00	0.93	
Limestone	77.10	75.20	0.98	
Marble	87.10	84.40	0.97	
Siltstone	71.30	65.00	0.91	
Tuff	15.50	13.70	0.88	
Calcareous mudstone	107.00	100.30	0.94	Liu et al. [28] (2013)
Monzonitic granite	208.41	186.39	0.89	Liang et al. [29] (2013)
Sandstone	67.58	51.32	0.76	Wei et al. [30] (2018)
Limestone	144.45	121.66	0.84	Ping et al. [31] (2018)
Sandstone	31.33	27.16	0.87	Jessu and Spearing [32] (2019)
Marble	155.60	126.40	0.81	Du et al. [33] (2019)

**Figure 1:**
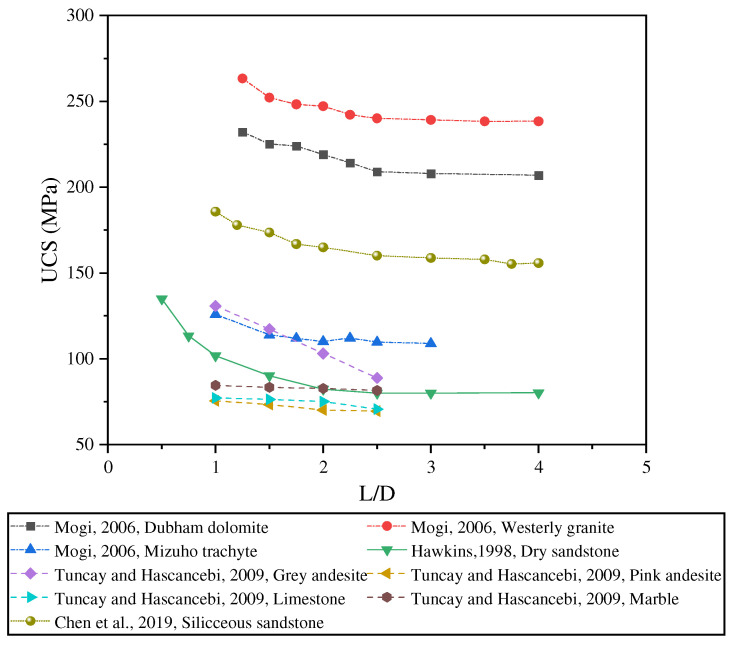
Relationship between UCS and L/D ratio [4,24,38,49].

The UCS of rocks typically decreases as the L/D ratio increases [3,4,13,34,35], a phenomenon corroborated by Liu et al. [36] and Yang et al. [37]. This effect is attributed to the heightened stress concentration caused by a larger L/D ratio, resulting in earlier crack initiation and accelerated propagation [38]. Figure 1 illustrates the relationship between the L/D ratio and rock mechanical properties as reported by various scholars, further validating the universality of the L/D ratio effect on UCS. Various standards propose differing recommendations for the minimum specimen diameter. The ISRM Suggested Methods [39] and ASTM [22] suggest minimum diameters of 54 mm and 47 mm, respectively. These values are very close to the preferred value of 50 mm proposed by Hoek and Brown [40] and are consistent with the value recommended by Hawkins [24]. When testing limestone, it is important to ensure that the L/D ratio of the specimen is greater than 1.0 to obtain optimal results [41]. Studies by Güneyli and Rüşen [12] and Tuncay et al. [5] confirm that the same trend applies to rock samples. In other words, as the specimen diameter increases, UCS increases [42].

Hodgson and Cook [43] and Obert et al. [44] reported that rock strength remains unaffected by changes in specimen size. In contrast, Mogi [45], Bieniawski [46], Pratt et al. [47], and Hoskins and Horino [48] reported that rock strength significantly decreases with an increase in specimen size. Hoek and Brown [40] measured the UCS of rock cores with diameters ranging from 10 mm to 200 mm. The data showed that UCS decreases with increasing specimen diameter. Using these data, they proposed a formula to convert the strength values of specimens with varying diameters to the standard strength value of a 50 mm diameter specimen, widely used in laboratories at the time.

(3) $$UCS=UCS_{D50}(\frac{50}{D}{)}^{0.18}$$

Hawkins [24] conducted uniaxial compression tests on various rock types, classifying them into igneous, sedimentary, and metamorphic rocks, with specimen diameters ranging from 12.5 mm to 150 mm. The igneous rock (Bath stone) showed stable and high compressive strength, with UCS values stabilizing as diameter increased, indicating minimal L/D ratio effects due to its uniform structure and high density. In contrast, sedimentary rocks, such as the Pillton Set and Clifton Down List, exhibited significant UCS fluctuations, with larger diameter cores showing stabilized UCS values as the influence of the L/D ratio diminished, suggesting that smaller cores are more affected by porosity and stratification. Metamorphic rocks (Hollington Set) displayed the smallest variation in UCS, with compressive strength stabilizing as core diameter increased, reflecting minimal L/D ratio effects due to their dense composition. The study concluded that the maximum strength for these rocks occurred in cores with diameters of approximately 40 to 60 mm, with UCS values for both larger and smaller diameter cores being relatively low. The findings of Hawkins [24] differ from those of Hoek and Brown [40].

Mogi [34,38] conducted laboratory tests on dolomite, granite, and rhyolite cores with L/D ratios ranging from 1.0 to 4.0. According to his findings, when the L/D ratio exceeds or equals 2.5, the UCS values remain relatively constant. For cores with an L/D ratio below 2.5, the UCS increases significantly. Hawkins [24] observed that specimens with an L/D ratio of 2.0 or greater exhibit relatively constant UCS values, whereas those with smaller ratios demonstrate a significant increase in UCS.

Obert et al. [44] and Obert and Duvall [35] observed that for cylindrical specimens, as long as the L/D ratio remains constant, the influence of specimen size on strength is less significant than the inherent variability of the rock itself. However, they also noted that the L/D ratio significantly affects the UCS, and this influence can be accounted for using the following relationship:

(4) $$UCS_{1}=UCS_{I}/(0.778+0.222D/L)$$

where *UCS*_1_ is the uniaxial compressive strength of a specimen with an L/D ratio of 1, and *UCS_I_* is the compressive strength of a specimen with an L/D ratio ranging from 1/3 to 2.0.

Tuncay et al. [4] conducted tests on seven distinct rock types, with specimen L/D ratios varying from 1.0 to 2.5. The study demonstrated that except for pumice, the UCS of all other rocks decreased as the L/D ratio increased to 2.5. The results further revealed that specimens with an L/D ratio below 2.0 exhibited relatively high UCS values, whereas the UCS remained nearly constant for ratios between 2.0 and 2.5. For specimens with an L/D ratio exceeding 2.5, UCS values stabilized. From the study’s findings, a correction formula was proposed to convert UCS values obtained at varying L/D ratios into those corresponding to a ratio of 2.5. However, this formula is limited to the seven rock types tested in the study, necessitating further research on other rock types for validation or refinement.

(5) $$UCS_{2.5}=UCS_{M}/[1.26-0.1(L/D)]$$

where *UCS*_2.5_ is the corrected uniaxial compressive strength corresponding to an L/D ratio of 2.5, and *UCS_M_* is the compressive strength measured on specimens with L/D ratios ranging from 1.0 to 2.5.

When a rock specimen reaches the UCS, a macroscopic fracture surface forms within the specimen, leading to failure and loss of UCS. Studies have shown that the L/D ratio significantly influences the failure modes of rocks [10], which exhibit various failure patterns, including block failure, conical failure, columnar splitting failure, and shear failure modes [11]. Specifically, the stress distribution and end-face friction effects [38] during loading of rock specimens with different L/D ratios directly affect their failure morphology.

1. Block failure mode (dense columnar splitting)

When the L/D ratio is small (L/D = 0.5), dense vertical cracks develop within the rock, causing the specimen to split into multiple blocks. Some blocks detach from the main body, resulting in numerous fragments, a characteristic feature of the block failure mode. This mode of failure is accompanied by loud noises and debris ejection, reflecting a strong release of elastic energy.

2. Conical failure mode

Conical failure typically occurs at small L/D ratios. In this failure mode, conical fractures form at both ends of the rock specimen due to end-face friction effects, which create compressive stress zones at the ends and tensile stress zones in the interior. Consequently, the sides of the rock specimen peel off, generating multiple rock fragments.

3. Columnar splitting failure mode

Columnar splitting failure is primarily a tensile failure caused by axial loading, which induces tensile stresses that exceed the rock’s tensile strength. Low tensile strength is the primary factor behind this mode, which predominantly occurs in specimens with smaller L/D ratios. In this failure mode, multiple parallel or nearly parallel splitting planes form along the axial direction of the specimen, exhibiting the characteristic features of columnar splitting.

4. Shear failure mode

At larger L/D ratios (L/D ≥ 2.0), the dominant failure mode is shear failure, characterized by a distinct directional pattern on the failure surface. Uneven stress distribution within the specimen leads to stress concentration zones, facilitating the propagation and coalescence of microcracks. These microcracks ultimately form a shear failure surface that penetrates the specimen. This mode becomes increasingly dominant as the L/D ratio increases.

As the L/D ratio of a rock specimen increases, the specimen exhibits a greater number of defects, such as pores and microcracks, leading to reduced homogeneity and lower strength. Under axial loading, uneven stress distribution within the specimen causes the formation of stress concentration zones. These zones facilitate the expansion, interconnection, and penetration of microcracks, ultimately resulting in the development of shear failure surfaces with distinct directional characteristics. In general, the failure modes of rock specimens with varying L/D ratios can be classified as brittle failures. For specimens with a small L/D ratio (L/D < 2.0), multiple splitting planes are likely to form along the axial direction, with splitting failure as the dominant mode. As the L/D ratio increases, the failure mode transitions to shear failure characterized by a single fracture surface. Overall, with increasing L/D ratios, the failure mode of rock specimens evolves from complex splitting failures to shear failures with a single rupture surface.

The L/D ratio significantly influences the energy evolution process of rocks. During loading, specimens with larger L/D ratios typically exhibit lower energy accumulation capacity. This phenomenon occurs because stress concentration causes the rock to reach the failure stage earlier, resulting in reduced elastic potential energy storage and increased plastic energy dissipation, thereby exhibiting higher ductility characteristics. Meng et al. [11] highlighted that as the L/D ratio increases, the rock’s capacity for energy accumulation and dissipation weakens, leading to a more concentrated energy release during failure and, consequently, more brittle failure characteristics. Similarly, Yan et al. [10] found that specimens with smaller L/D ratios have a higher energy storage capacity. With increasing L/D ratios, a greater portion of input energy is converted into dissipated energy, resulting in failure accompanied by more plastic deformation. Gong et al. [6] validated the energy evolution trends for different L/D ratios, finding that regardless of the specimen diameter, an increase in the L/D ratio leads to a higher proportion of elastic energy and a lower proportion of dissipated energy, reflecting a significant change in energy storage and dissipation characteristics. Overall, as the L/D ratio increases, rocks tend to accumulate more elastic energy during loading while exhibiting reduced energy dissipation, a behavior that directly impacts their failure mode and stability.

### 2.2. The Length-to-Diameter Ratio Effect in Triaxial Compression Tests

In triaxial compression tests, rock strength generally increases with confining pressure. However, an increase in the L/D ratio suppresses this strengthening effect.

**Figure 2:**
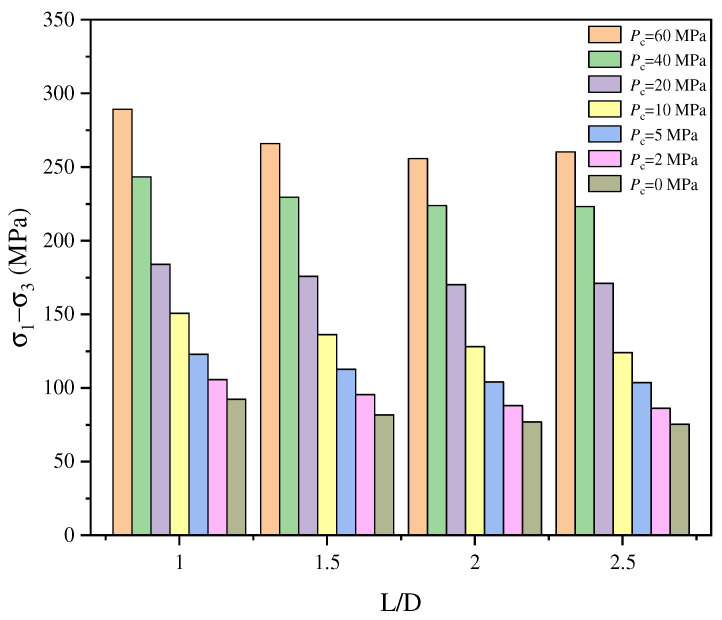
Comparison of TCS of siltstone specimens with different L/D ratios under different confining pressures [50].

Zhu et al. [50] performed triaxial compression tests on sandstone specimens with identical diameters but varying heights. The results revealed that under identical confining pressure, the compressive strength of sandstone decreases with increasing L/D ratio. Notably, when the L/D ratio exceeds 2.2, the mechanical properties of sandstone stabilize, as illustrated in Figure 2. These findings underscore a complex interplay between the L/D ratio and confining pressure, collectively influencing the compressive strength of rock.

**Figure 3:**
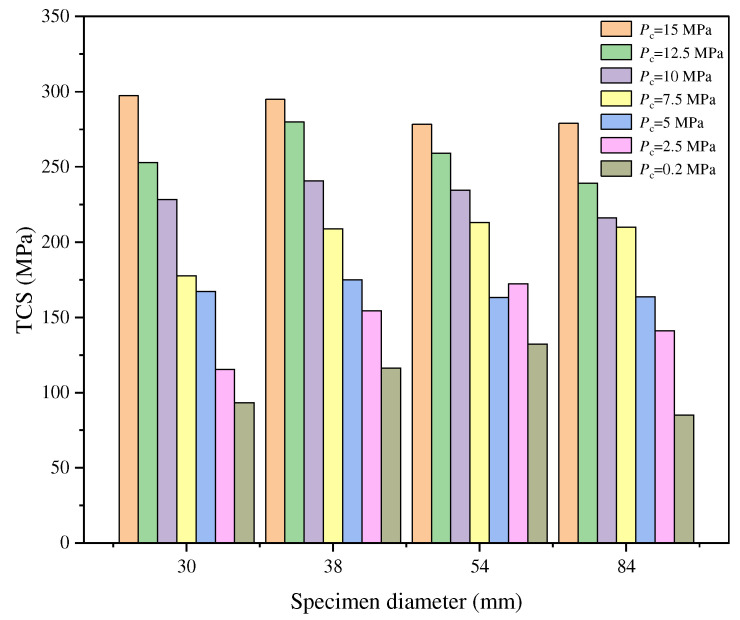
Relationship between TCS and diameter of granite specimens under different confining pressures [51].

Manuel et al. [51] conducted several triaxial compression tests on granite specimens with diameters ranging from 30 to 84 mm, while maintaining a constant L/D ratio, and subjected them to confining pressures ranging from 0.2 to 15 MPa. The results indicated that under low confining pressures, triaxial compressive strength (TCS) initially increases before decreasing as specimen diameter increases. However, under high confining pressures, this trend becomes less pronounced, as shown in Figure 3. This phenomenon occurs because high confining pressures promote the closure of microcracks, reducing the influence of specimen diameter on TCS. These findings are consistent with the conclusions of Aubertin et al. [52].

**Figure 4:**
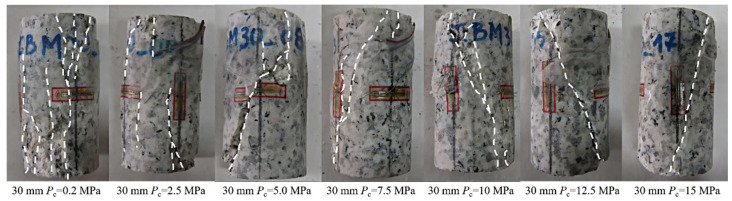
Failure patterns of granite specimens with 30 mm diameter under different confining pressures (Red squares represent strain gauges; white dashed lines indicate crack propagation directions) [51].

The energy characteristics of rock specimens are significantly influenced by the L/D ratio during triaxial compression loading. Specimens with smaller L/D ratios accumulate more elastic energy during loading, which is rapidly released upon failure. In contrast, specimens with larger L/D ratios dissipate more plastic energy, resulting in a more gradual failure process and greater ductility. Zhu et al. [50] demonstrated that rock specimens with larger L/D ratios undergo greater energy dissipation under confining pressure, indicating that these specimens resist external loads primarily through plastic deformation during failure. Therefore, an increase in the L/D ratio alters the energy storage and release mechanisms of the rock, directly influencing its failure behavior and deformation characteristics. Under low confining pressure, the failure mode is predominantly axial splitting, while higher confining pressures lead to a transition toward shear failure. Furthermore, Manuel et al. [51] confirmed that under low confining pressures, failure modes are predominantly axial splitting, while at higher confining pressures, failure gradually transitions to shear band formation (Figure 4).

### 2.3. The Length-to-Diameter Ratio Effect in Static Tensile Tests

In static tensile tests, the tensile strength of rock specimens is typically determined using two main methods: the direct tensile test (DTS) and the Brazilian tensile test (BTS).

DTS typically uses cylindrical specimens under pure tensile loading, resulting in uniform tensile stress. To date, studies on the influence of L/D ratios on DTS or uniaxial tensile strength in rocks remain limited. A pioneering study by Obert et al. [44] explored the effect of specimen diameter on tensile strength and represented an early effort to standardize uniaxial tensile strength testing methodologies. Wijk et al. [53] investigated the effect of specimen size on the uniaxial tensile strength of Bohus granite and concluded that the tensile strength dependence on specimen size is negligible for cylindrical specimens with diameters ranging from 8 mm to 62 mm and lengths of d, 2d, and 4d. Van Vliet and Van Mier [52], in testing dog-bone-shaped specimens of varying sizes made of sandstone, observed an increase in tensile strength with specimen size. Coviello et al. [54] conducted an extensive study on the tensile strength of soft rocks, performing DTS tests on dried calcareous sandstone specimens with diameters of 25 mm, 38 mm, 50 mm, and 60 mm. They found no significant correlation between specimen size and tensile strength. Jensen [55] analyzed the relationship between specimen size and tensile strength for other sedimentary rocks, conducting DTS tests on Castlegate sandstone and Mons chalk. The results showed that the tensile strength of sandstone increased with specimen diameter, while that of chalk exhibited the opposite trend. However, this study considered only two different diameters.

BTS typically involves Brazilian splitting, in which compressive stress is applied to induce tensile stress within the specimen. In Brazilian splitting tests, the L/D ratio significantly affects the tensile properties and splitting strength of rock materials. By varying the specimen’s L/D ratio, one can observe changes in the rock’s splitting strength as well as the evolution of cracks during the failure process. The L/D ratio not only influences the splitting strength but also affects the initiation point and propagation path of cracks, thereby determining the differences in failure modes observed in the test.

**Figure 5:**
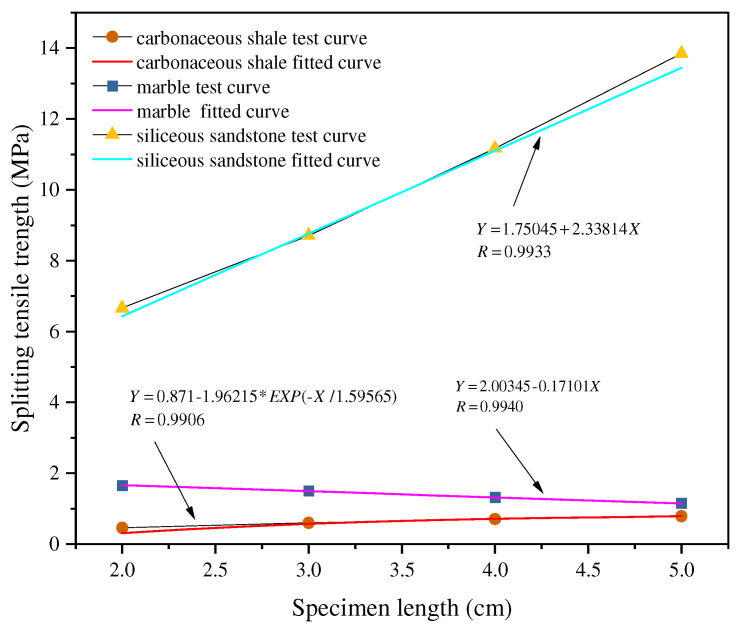
Relationship between specimen length and splitting tensile strength [58].

Generally, as the L/D ratio increases, the tensile strength of rocks tends to decrease. Su [56] pointed out that the tensile strength of red sandstone significantly decreases with increasing L/D ratio, which is attributed to the enhanced stress concentration effect caused by the higher L/D ratio, making cracks more susceptible to initiation and propagation, thereby reducing the overall tensile strength. Yin et al. [57] further confirmed this phenomenon, showing that tensile strength decreases cubically as the L/D ratio increases. Xu et al. [58] prepared specimens of marble, siliceous coarse sandstone, and carbonaceous mudstone with consistent diameters (50 mm) but varying thicknesses (20–50 mm). Their analysis revealed that the specimen thickness affected the splitting tensile strength, with rocks having higher UCS showing greater sensitivity of the Brazilian splitting strength to changes in the L/D ratio. Interestingly, their analysis indicated that the trend of splitting strength with dimensional change varied by rock type, contrary to the conventional view that strength decreases with increasing specimen size. Specifically, the splitting strength of marble decreased linearly with increasing thickness, while that of siliceous sandstone increased linearly with thickness, and carbonaceous mudstone exhibited an exponential increase in splitting strength with increasing thickness, as shown in Figure 5.

**Figure 6:**
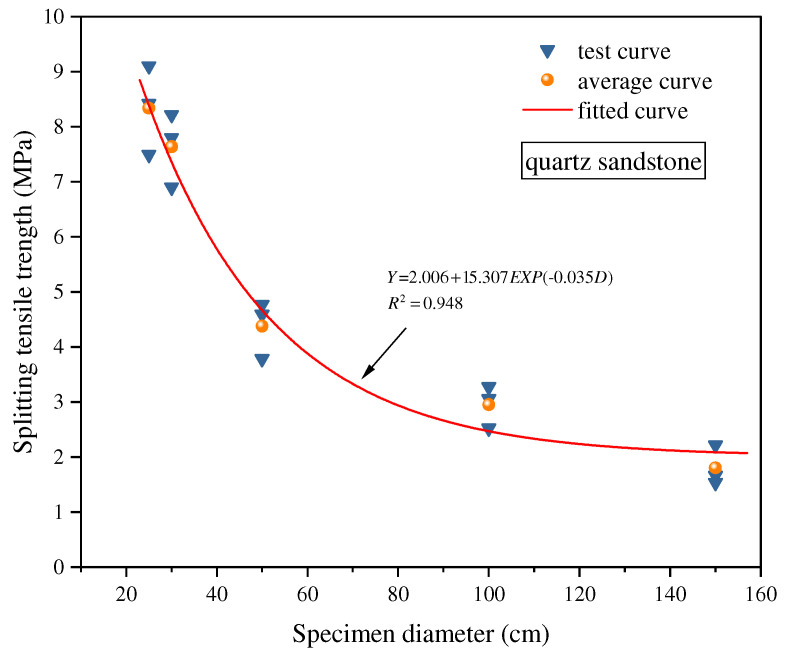
Relationship between specimen diameter and splitting tensile strength [59].

These studies primarily focused on the effect of variations in specimen thickness. Xu et al. [59] further pointed out that under a constant L/D ratio, when the specimen diameter increased from 25 mm to 150 mm, the corresponding tensile strength initially decreased rapidly, then more gradually, stabilizing once the diameter exceeded 50 mm. The relationship between specimen diameter and splitting tensile strength is depicted in Figure 6. Furthermore, Alawad [60] proposed an improved formula for Brazilian splitting tensile strength to better accommodate tests on specimens with varying L/D ratios. The results indicated that an increase in L/D ratio led to a decrease in tensile strength, and the revised formula more accurately reflected this trend, significantly improving the accuracy of the experimental results. He et al. [61] improved the measurement method using digital image correlation technology and found that increasing the L/D ratio not only gradually reduced tensile strength but also had a significant impact on the crack initiation point and propagation path.

The L/D ratio also significantly affects the splitting failure modes of rock specimens. Studies have shown that rock specimens with varying dimensions exhibit distinct failure patterns in splitting tests, highlighting the strong correlation between failure modes and geometric properties [62,63]. Wei et al. [62] investigated the failure patterns of granite and sandstone specimens by varying their L/D ratios while maintaining constant specimen thickness. Their findings revealed consistent failure characteristics across specimens of varying sizes: the fracture plane typically aligned with the centerline of the loading pads, dividing the specimens into two nearly symmetrical halves. Specimens with central cracking exhibited rapid crack propagation: cracks initiated at the center, extended swiftly toward the stress concentration points, and eventually traversed the cross-section, splitting the specimen into two symmetric halves.

The tensile strength and failure modes of coal and rock under tensile stress exhibit significant variation depending on the rock type [64,65]. Generally, three primary tensile failure modes are identified: (1) central failure, characterized by a dominant crack located at the disk’s center, approximately parallel to the loading direction [66]; (2) non-central failure, involving a dominant arcuate crack that crosses the peripheral region of the disk specimen [65]; and (3) combined central failure, wherein at least one major crack traverses the central region, accompanied by additional side cracks [67].

Song et al. [63] investigated the tensile failure modes of coal specimens with a constant L/D ratio but varying diameters. Four distinct failure patterns were identified: central failure, non-central failure, central-edge failure, and central-multiple failure. Central and non-central failure modes were observed across various coal types, closely resembling the failure modes of other rocks such as sandstone [64], granite [65], and shale [66]. Notably, coal exhibited unique failure patterns, namely central-edge failure and central-multiple failure. Central-edge cracking initiated at a loading point, propagated through the disk’s center, and bifurcated near the opposite loading point. In contrast, central-multiple cracking typically involved a primary crack traversing the specimen, interconnected with or interrupted by several secondary cracks.

## 3. Influence of Length-to-Diameter Ratio on Dynamic Mechanical Properties of Rocks

In rock dynamics research, the L/D ratio plays a critical role in determining the dynamic mechanical response of rocks. Through SHPB tests, researchers have investigated the influence of the L/D ratio on the dynamic mechanical properties of rocks.

### 3.1. The Length-to-Diameter Ratio Effect in SHPB Uniaxial Compression Tests

**Figure 7:**
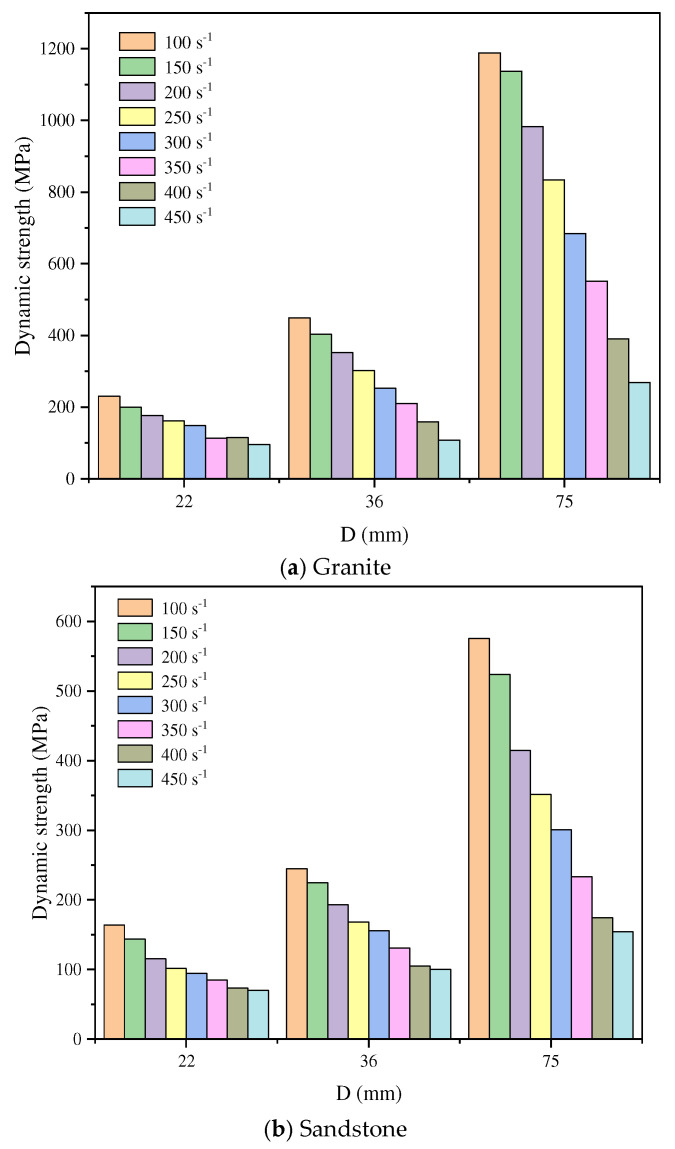

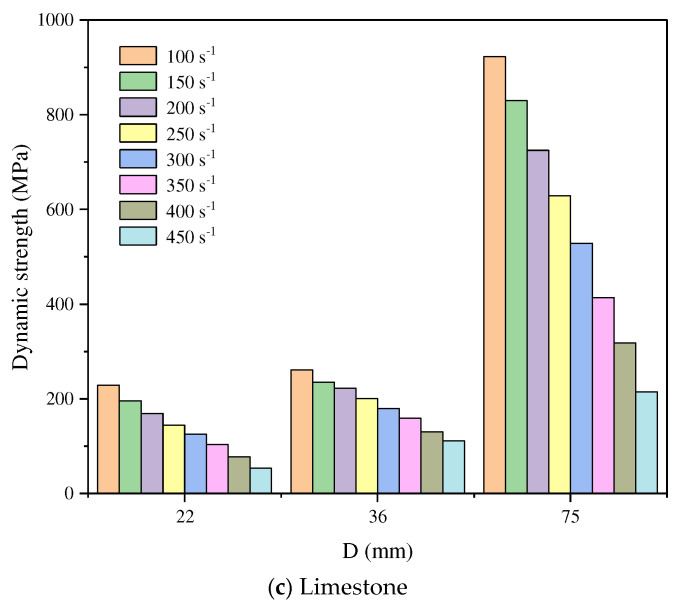
Size effect of rock strength under different strain rates [68].

In dynamic uniaxial compression tests, the rock strength varies significantly with changes in the L/D ratio. Generally, an increase in the L/D ratio extends the stress wave propagation path within the specimen, causing greater energy attenuation and ultimately reducing the strength. Hong et al. [68] performed SHPB tests on granite, sandstone, and limestone specimens with a fixed L/D ratio of 0.5 and diameters of 22, 36, and 75 mm under varying strain rate conditions. The results revealed a positive correlation between the dynamic UCS of the rocks and the strain rate. Additionally, larger specimen sizes amplified the sensitivity of dynamic UCS to strain rate. Under identical strain rates, the dynamic UCS of rocks increased with specimen size (Figure 7), which contrasts with the size effect observed under static loading conditions [16]. As shown in Figure 7, although variations exist in the specific results for the three rock types, the general trend is consistent: higher strain rates amplify the effect of specimen size on strength, whereas lower strain rates diminish this effect. This suggests the existence of a critical strain rate beyond which the size effect on rock strength vanishes. Below this threshold, static size effects dominate, while above it, dynamic size effects prevail. Kao et al. [69] further confirmed the significant correlation between the L/D ratio and strain rate.

In SHPB tests, the dynamic UCS of sandstone specimens varied significantly with increases in both the L/D ratio and strain rate, following a cubic relationship between dynamic UCS and L/D ratio. Strength analysis of rock specimens under various L/D ratios and strain rates revealed that an increase in specimen length had a significantly greater impact on dynamic UCS than variations in strain rate, especially at higher impact velocities. These findings highlight the critical role of specimen size in determining the mechanical properties of rocks under dynamic loading conditions. Based on experimental results, the authors recommend selecting an optimal L/D ratio (between 0.5 and 0.8) to achieve stable and predictable strength characteristics of rock specimens under dynamic loads. Huang et al. [70] investigated the dynamic mechanical properties of granite specimens with a fixed L/D ratio (0.5) but varying diameters (50, 60, and 150 mm) under high strain rates. They observed that the dynamic uniaxial strength increased with specimen diameter under the same strain rate. Zou et al. [71,72] examined the influence of specimen size and geometry on the dynamic mechanical properties of Carrara marble and discovered that the dynamic brittleness index was also affected by the L/D ratio.

The effect of the L/D ratio on rock failure modes during dynamic processes is multifaceted. Research indicates that an increase in the L/D ratio leads to greater complexity in rock failure modes, significantly altering crack initiation, propagation, and ultimate fracture patterns. Rock specimens with higher L/D ratios typically generate more fine fragments under dynamic loading, attributed to crack refinement and the development of a denser crack network. The crack propagation paths become increasingly intricate with higher L/D ratios, resulting in a pronounced increase in failure mode anisotropy.

**Figure 8:**
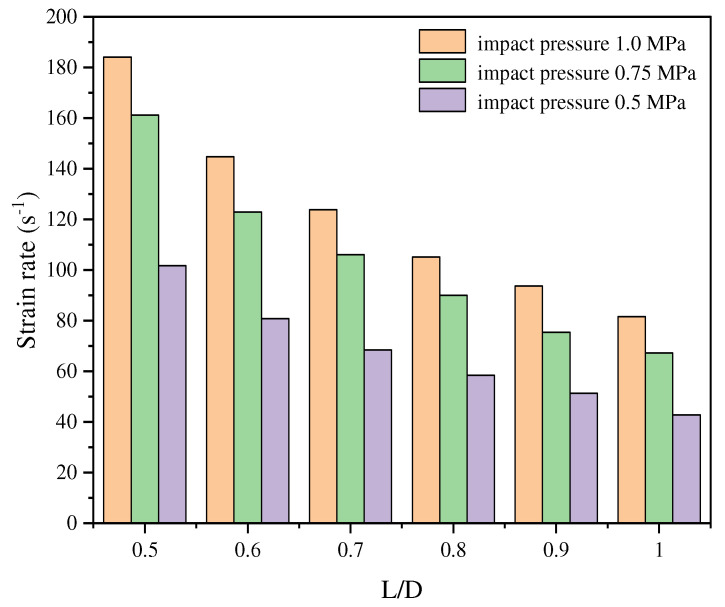
Relationship between strain rate and size [15].

From a microscopic perspective, the L/D ratio significantly influences crack initiation and propagation. A higher L/D ratio accelerates crack propagation along stress concentration regions, generating finer fragments and thereby influencing the dynamic UCS of the rock. Zhou et al. [15] observed that at the same strain rate, larger specimens tend to generate finer and more abundant fragments. The relationship between strain rate and specimen size is illustrated in Figure 8. At the same strain rate, larger specimens are associated with higher impact loads; this means that more energy is available for fracture, resulting in smaller fragment sizes. Under identical impact conditions, the strain rate decreases with increasing specimen size.

**Figure 9:**
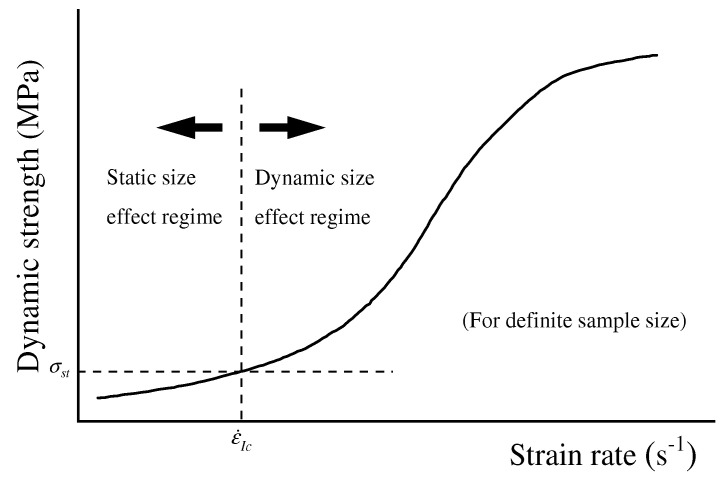
The characteristic transition strain rate [73].

The initial stage of crack propagation is primarily influenced by the distribution of micro-cracks within the rock and its geometric dimensions. Consequently, an increase in the L/D ratio directly affects the initiation and propagation paths of cracks. This phenomenon is critically important to the dynamic failure process of rocks, as it governs energy release during failure and the extent of resulting damage. Qi et al. [73,74,75] provided a theoretical explanation of the underlying mechanisms of this phenomenon. Their study, based on the Maxwell relaxation model, examined how limited crack propagation speed affects the dynamic loading process. When the strain rate surpasses the characteristic strain rate, dynamic loading dominates (Figure 9), and limited crack propagation speed intensifies stress concentration. As the L/D ratio increases, the time required for cracks to propagate within the rock extends significantly, elevating stress levels prior to macroscopic failure and altering the failure mode. The Maxwell relaxation model highlights how limited crack propagation speed impacts the failure mechanism of rocks under dynamic loading, particularly with varying L/D ratios. Under impact loads, crack propagation speed is inherently finite, resulting in localized stress concentration during propagation. In specimens with larger L/D ratios, crack penetration takes longer, leading to greater stress accumulation. This delayed failure process at elevated stress levels is a key factor in the reduction of macroscopic strength and fragment refinement.

An increase in the L/D ratio affects not only the dynamic modulus of rocks but also their energy absorption capacity significantly. A larger L/D ratio leads to earlier crack initiation and more rapid propagation at higher stress levels, enhancing the specimen’s energy absorption capacity. This difference in energy absorption is particularly evident during the deformation of schist specimens [16], as their complex internal structure significantly influences crack propagation paths. Increasing the L/D ratio amplifies the complexity of crack propagation paths, resulting in greater energy dissipation. Specimens with smaller L/D ratios store more elastic energy and experience less energy dissipation, whereas larger ratios result in greater plastic energy dissipation, characterized by extensive crack propagation and ductile deformation.

### 3.2. The Length-to-Diameter Ratio Effect in SHPB Triaxial Compression Tests

**Figure 10:**
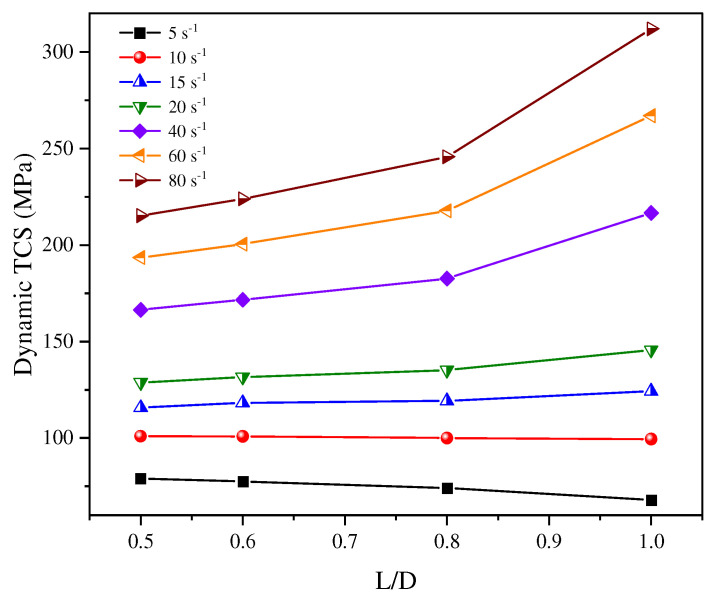
Relationship between dynamic TCS and L/D ratio at different strain rates.

Compared to uniaxial loading, the stress state under triaxial stress conditions better represents real-world engineering environments. The strength of rock materials typically varies with changes in confining pressure and dynamic strain rate, with the L/D ratio playing a critical regulatory role in this behavior. Research on the size effect of dynamic TCS remains limited. Wu et al. [76] investigated the dynamic TCS of coal samples with diameters of 50 mm and four different L/D ratios. Their experimental results indicated that under the same strain rate, the dynamic triaxial compressive strength of the coal samples increased with an increasing L/D ratio, consistent with findings from dynamic uniaxial compression tests. Similarly, Luo et al. [7] emphasized that in dynamic compression experiments, a larger L/D ratio results in a longer crack propagation time within the specimen. Furthermore, it was observed that at varying strain rates, the sensitivity of the peak stress to the L/D ratio effect changes. The influence of the L/D ratio becomes more pronounced with increasing strain rates (as shown in Figure 10).

**Figure 11:**
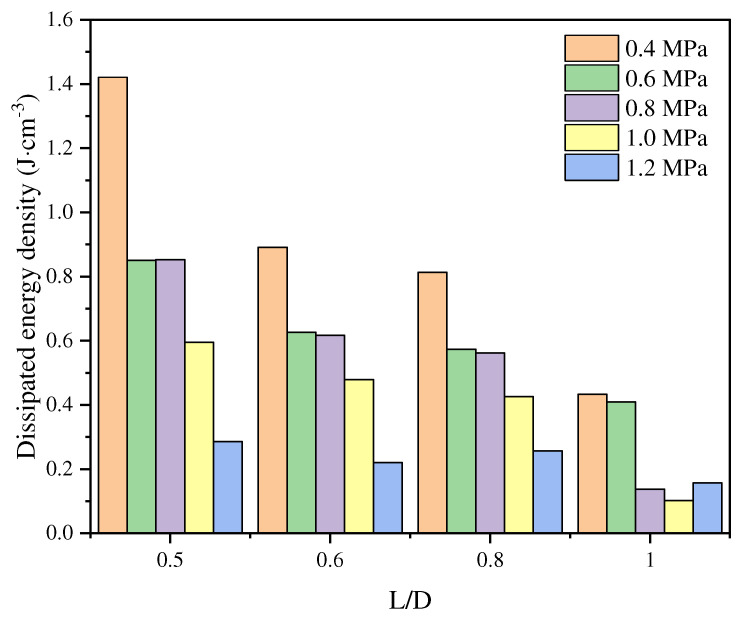
Relationships between dissipated energy density and L/D ratio [76].

Under dynamic loading conditions, the L/D ratio significantly influences the failure modes and energy dissipation characteristics of specimens. As illustrated in Figure 11, at varying pressure levels (ranging from 0.4 MPa to 1.2 MPa), the fracture energy density of the specimens decreases with an increasing L/D ratio. This observation indicates that under identical pressure conditions, specimens with larger L/D ratios dissipate less energy per unit volume, resulting in lower fracture energy density. The trend becomes more pronounced at higher pressures, such as 1.2 MPa. This behavior can be attributed to the enhanced structural stability provided by a larger L/D ratio during impact, which reduces the energy dissipation per unit volume.

The failure modes of specimens vary significantly with changes in pressure levels and L/D ratios. At lower pressure levels (e.g., 0.4 MPa), specimens predominantly exhibit minor damage characterized by a few surface cracks. However, as the pressure increases to 0.8 MPa and above, the failure mode transitions from slight damage to severe fragmentation, particularly in specimens with smaller L/D ratios. This indicates that smaller L/D ratios result in a higher susceptibility to severe fragmentation under elevated pressures, whereas specimens with larger L/D ratios generally retain relatively intact failure modes, even under high-pressure conditions. The observed differences in failure modes under identical pressure conditions can be attributed to variations in structural characteristics and stress distribution following loading.

Additionally, a clear correlation exists between strain rate and L/D ratio. At a constant pressure level, specimens with smaller L/D ratios generally exhibit higher strain rates, while those with larger ratios display lower strain rates. This suggests that specimens with smaller L/D ratios experience more intense deformation during impact, leading to more severe fragmentation. Furthermore, as the pressure increases, the strain rates of all specimens, regardless of their L/D ratios, also rise, aligning with the intensity of the observed failure modes.

### 3.3. The Length-to-Diameter Ratio Effect in Dynamic Tensile Tests

Dynamic tensile tests are crucial for exploring the tensile strength and failure characteristics of rocks under dynamic conditions. Despite their importance, research on the effect of specimen size on the dynamic mechanical properties of rocks has predominantly focused on compressive behavior, with limited attention to tensile properties. Jia et al. [17] compared dynamic direct and indirect tensile strengths using the split Hopkinson tensile bar (SHTB) and SHPB systems, emphasizing the influence of specimen size (L/D ratio) on the tensile properties of rock materials. The findings reveal that the size of the specimen significantly affects both direct and indirect tensile strengths under dynamic loading.

**Figure 12:**
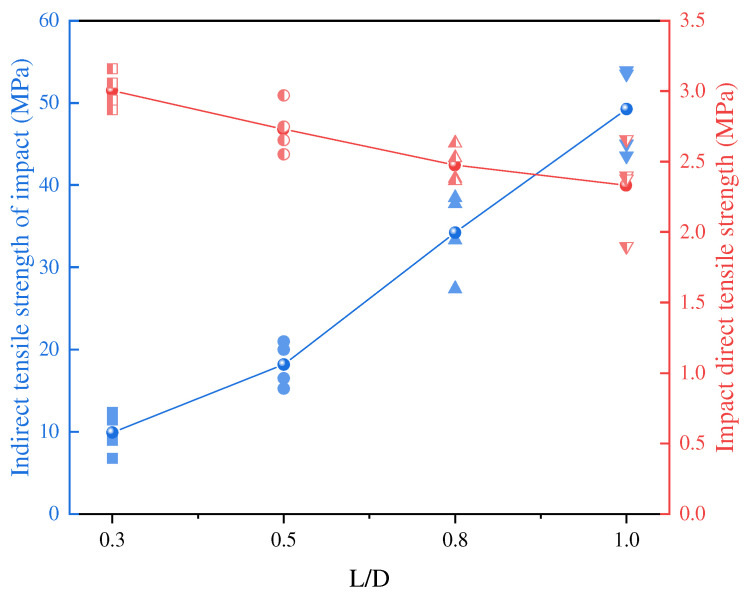
Dynamic direct tensile and indirect tensile strength of specimens with different L/D ratios [17].

In dynamic direct tensile tests, tensile strength generally decreases with increasing L/D ratio. Larger specimens exhibit more internal fractures, microcracks, and structural heterogeneities, which accelerate crack propagation and lead to earlier failure under identical tensile loads. Conversely, in dynamic indirect tensile tests, the effect of the L/D ratio is more pronounced and exhibits an opposite trend (Figure 12). With increasing L/D ratio, indirect tensile strength increases significantly due to the distribution of applied forces over a larger area, reducing localized stress concentrations.

Additionally, the failure modes in dynamic direct and indirect tensile tests show distinct differences. In dynamic direct tensile tests, specimens with a smaller L/D ratio typically display brittle tensile failure. Cracks propagate rapidly along the direction of the applied tensile force, and failure is concentrated in the central portion of the specimen. This process occurs rapidly and without significant plastic deformation. As the L/D ratio increases, the failure mode gradually shifts to shear failure. In this case, cracks no longer propagate solely in the tensile direction but deviate from the central region, forming fracture planes that intersect the upper and lower surfaces of the specimen at an angle. This shear failure characteristic indicates that larger specimens experience more complex stress distributions, and the internal stress and crack propagation patterns change accordingly.

In dynamic indirect tensile tests, smaller specimens often exhibit localized crushing failure, with damage concentrated at the ends, forming distinct triangular crushing zones. As the L/D ratio increases, the failure mode becomes more uniform, with the crushed zone area gradually decreasing, and the damage process becomes more dispersed and steady. This change suggests that larger specimens can effectively distribute the applied external stress, thereby reducing the risk of local stress concentrations. This results in a more uniform failure process and enhanced overall resistance to failure.

Overall, size effects significantly influence failure modes in dynamic tensile tests. Notably, in both direct and indirect tensile responses, an increase in specimen size causes the rock failure process to transition from localized, brittle failure to more uniform and complex failure modes.

## 4. Perspective on the Length-to-Diameter Ratio Effect

Although considerable progress has been achieved in understanding the L/D ratio effect in rocks, several critical challenges remain unresolved. Future research should focus on elucidating the micromechanical mechanisms, advancing multi-scale modeling methodologies, and investigating the influence of complex environmental factors. These efforts are essential for providing more accurate theoretical foundations for rock mechanics and delivering practical insights for engineering applications.

### 4.1. Micromechanical Mechanisms Underlying the Length-to-Diameter Ratio Effect

The mechanisms underlying the L/D ratio effect in rock mechanics remain a significant research challenge. Experimental studies and theoretical analyses have demonstrated the influence of the L/D ratio on rock strength, fracture patterns, and microstructural evolution [6,10,77]. However, the precise micromechanical mechanisms governing these effects are still not well understood. In particular, the role of the L/D ratio in controlling crack propagation, fracture processes, and the evolution of both macro- and microstructures within rocks requires further detailed investigation [7]. At the microscale, the L/D ratio critically affects the behavior of microcracks, influencing their initiation, propagation, and eventual coalescence [14]. Rocks with different L/D ratios exhibit distinct crack propagation paths and failure modes under external loading [26]. These microscopic alterations not only modify the rock’s strength and toughness but also significantly impact its stability under high-stress conditions.

Future research should prioritize the study of crack propagation through various rock structures, such as pores and mineral interfaces, under different L/D ratios. Furthermore, it is essential to explore how these microstructural transformations translate into macroscopic mechanical behaviors, thereby providing deeper insights into the fundamental nature of the L/D ratio effect. Such findings will be crucial for refining theoretical models in rock mechanics and advancing their application in engineering practices.

### 4.2. Multi-Scale Modeling and Numerical Simulation

With the continuous advancements in computer science and mechanical simulation techniques, multi-scale modeling has emerged as a crucial tool for investigating the L/D ratio effect in rocks. By bridging macroscopic experimental observations and microscopic mechanisms, this approach enables researchers to make more accurate predictions of the mechanical behavior of rocks in complex environmental conditions. As research progresses, multi-scale numerical simulations are expected to play an increasingly vital role in uncovering the intricate relationships between micro- and macro-scale processes. By leveraging advanced numerical simulation techniques, such as the FEM and DEM, researchers can conduct detailed analyses of crack propagation behavior under varying L/D ratio conditions [37,78]. These simulations provide valuable insights into how crack evolution affects the macroscopic mechanical responses of rocks [79,80,81]. A critical direction for future work involves seamlessly integrating microscale crack development mechanisms with macroscale mechanical models, particularly under complex stress states and loading conditions.

Furthermore, these integrated models can elucidate the influence of different L/D ratios on the mechanical response, failure modes, and stability of rocks [20]. By offering a more robust theoretical foundation, they hold the potential to significantly enhance the engineering applications of rock materials. Such advancements will not only improve the predictive accuracy of multi-scale models but also provide practical guidance for addressing engineering challenges in rock mechanics [82,83].

### 4.3. Investigation of the Length-to-Diameter Ratio Effect Under Complex Environments

The application of rock materials in practical engineering often requires consideration of complex environmental conditions, including high temperatures, high pressures, and chemical erosion [7,84]. These environmental factors may interact with the L/D ratio effect, resulting in highly intricate mechanical behaviors. Although previous studies have investigated the mechanical properties of rocks under extreme environments [56,85,86], the combined influence of the L/D ratio effect and these environmental conditions remains insufficiently explored. For example, in engineering fields such as deep-earth mining, geothermal energy development, and oil and gas drilling, understanding the interaction between environmental factors and the L/D ratio effect is critical. Future research should focus on how these complex conditions affect rock strength, crack propagation, and failure modes under varying L/D ratios. A significant technical advancement in this regard is the world’s first dynamic true-triaxial electromagnetic Hopkinson bar testing system, independently developed by Academician He-Ping Xie’s research team at Shenzhen University [87]. This cutting-edge system enables dynamic true-triaxial loading experiments on specimens with varying L/D ratios, providing an innovative approach for systematically investigating how the L/D ratio influences rock mechanical behavior under complex stress states.

### 4.4. The Length-to-Diameter Ratio Effect Analysis Driven by Machine Learning and Big Data

In recent years, data-driven methodologies, such as machine learning (ML) and deep learning, have rapidly advanced and found widespread applications across various engineering disciplines [88,89,90,91]. These technologies offer unprecedented capabilities to analyze the intricate relationships between the L/D ratio effect in rocks and other influential factors, including porosity, mineral composition, and environmental conditions, through big data. As research in rock mechanics continues to evolve, the integration of experimental data with artificial intelligence (AI) techniques is poised to provide more efficient and accurate tools for investigating the L/D ratio effect in depth.

Machine learning, in particular, has the potential to revolutionize how predictive models are developed based on extensive experimental datasets. By leveraging these models, researchers can uncover intrinsic relationships between the L/D ratio and key rock properties, such as strength, deformation behavior, and failure mechanisms. Data-driven analyses of the mechanical responses of rock samples under varying L/D ratio conditions not only enhance the accuracy and efficiency of predictions but also establish a scientific basis for the design and optimization of rock materials tailored to specific engineering needs.

Moreover, big data analytics, in conjunction with ML algorithms, enables the identification of hidden patterns and complex interactions in rock behavior under diverse conditions. This approach allows for the development of personalized and context-specific solutions for real-world engineering applications. By uncovering dynamics that traditional methods often overlook, data-driven techniques significantly advance the understanding of the L/D ratio effect and its implications for rock mechanics and material design, paving the way for more robust and innovative engineering practices.

## 5. Conclusions

This study investigates the influence of the L/D ratio on the mechanical properties, failure characteristics, and experimental outcomes of rocks. The results clearly demonstrate that the L/D ratio significantly affects rock mechanical performance. Specifically, an appropriate L/D ratio minimizes size effects and stress concentrations, thereby ensuring that experimental results accurately reflect in situ mechanical behavior. In static mechanical tests, such as uniaxial compression, triaxial compression, and static tensile tests, the L/D ratio was shown to influence compressive strength, tensile strength, and elastic modulus. Under high confining pressures, it also critically affects stress-strain behavior and failure modes, underscoring its importance in experimental design. In dynamic tests using the SHPB, the L/D ratio governs failure mechanisms and energy absorption under high strain rates. These findings highlight the need to optimize specimen dimensions for dynamic loading scenarios, ensuring that laboratory experiments remain relevant to real-world engineering conditions.

From an engineering perspective, selecting a rational L/D ratio, tailored to specific project requirements, plays a key role in improving the reliability of experimental data. This enables accurate mechanical parameters to be derived for design and safety assessments in practical engineering projects. Notably, this research offers vital experimental data that support rock mass stability analysis, tunnel construction, and deep foundation design, contributing to safer and more efficient engineering solutions. The appropriate choice of L/D ratio is particularly critical in extreme conditions, such as high confining pressure and dynamic loading, where it significantly influences the mechanical behavior and failure modes of rocks. Therefore, optimal L/D ratio selection is fundamental to ensuring the safety, stability, and cost-effectiveness of engineering projects.

Future research should focus on exploring the micromechanical mechanisms behind the L/D ratio effect, developing multi-scale modeling techniques, and investigating how L/D ratios interact with complex environmental factors, such as high temperatures and pressures. The integration of machine learning and big data analytics holds the potential to advance predictive modeling, providing deeper insights into the effects of the L/D ratio. Despite the progress made, challenges persist in obtaining consistent experimental results due to the inherent complexity of the L/D ratio effect. Further refinement is needed in the integration of experimental data with numerical simulations under extreme conditions. Addressing these challenges will enhance our understanding of the L/D ratio effect and facilitate further advancements in both rock mechanics theory and engineering practice.

## References

[1] Cai M.F., He M.C., Liu D.Y. (2002). Rock Mechanics and Engineering.

[2] Zhao Y.S. (2021). Retrospection on the development of rock mass mechanics and the summary of some unsolved centennial problems. Chin. J. Rock Mech. Eng..

[3] Pan P.Z., Feng X.T., Hudson J.A. (2009). Study of failure and scale effects in rocks under uniaxial compression using 3D cellular automata. Int. J. Rock Mech. Min. Sci..

[4] Tuncay E., Hasancebi N. (2009). The effect of length to diameter ratio of test specimens on the uniaxial compressive strength of rock. Bull. Eng. Geol. Environ..

[5] Tuncay E., Özcan N.T., Kalender A. (2019). An approach to predict the length-to-diameter ratio of a rock core specimen for uniaxial compression tests. Bull. Eng. Geol. Environ..

[6] Gong F.Q., Ni Y., Jia H. (2022). Effects of Specimen Size on Linear Energy Storage and Dissipation Laws of Red Sandstone under Uniaxial Compression. Bull. Eng. Geol. Environ..

[7] Luo K., Wang Y.M., Luo S., Li X.S., Peng K. (2024). Dynamic compressive behavior of impact-damaged and water-soaked sandstone with different length-to-diameter ratios. Arch. Civ. Mech. Eng..

[8] Pan J., Zhong W., Wang J., Zhang C. (2022). Size Effect on Dynamic Splitting Tensile Strength of Concrete: Mesoscale Modeling. Cem. Concr. Compos..

[9] Chen Y., Guo B. (2020). Crack Closure Effect and Energy Dissipation Model for Rocks under Uniaxial Compression. Geotech. Geol. Eng..

[10] Yan J., Gong F.Q., Luo S. (2022). Effects of length-to-diameter ratio on energy storage characteristics of rock materials under uniaxial compression. Bull. Eng. Geol. Environ..

[11] Meng Q., Liu J., Huang B., Zhang Z., Long J., Wu J. (2023). Experimental Analysis of the Height–Diameter Ratio Effect of Rock Energy under Uniaxial Cyclic Loading–Unloading Conditions. Bull. Eng. Geol. Environ..

[12] Güneyli H., Rüşen T. (2016). Effect of length-to-diameter ratio on the unconfined compressive strength of cohesive soil specimens. Bull. Eng. Geol. Environ..

[13] Thuro K., Plinninger R., Zäh S., Schütz S. (2001). Scale effects in rock strength properties. Part 1: Unconfined compressive test and Brazilian test. Rock Mechanics—A Challenge for Society.

[14] Chen Z.H., Liu C.W., Deng J.W. (2012). Size Effect of Uniaxial Compressive Strength of Limestone. Appl. Mech. Mater..

[15] Zhou J., Zhao G., Meng X., Liu C., Ma L., Xu W., Cheng X. (2023). Experimental Investigation of the Size Effect of Rock under Impact Load. Minerals.

[16] Mishra S., Khetwal A., Chakraborty T., Basu D. (2022). Effect of Loading Characteristics and Specimen Size in Split Hopkinson Pressure Bar Test on High-Rate Behavior of Phyllite. Arch. Civ. Mech. Eng..

[17] Jia Y., Zhai Y., Liu S. (2024). Study on the Size Effect of Rock under Dynamic Direct Tension and Indirect Tension. Environ. Earth Sci..

[18] Wang Y.M., Luo K., Peng K., Wu Q.H., Luo S., Ma T.X., Wu T., Yin X.Y. (2025). Experimental Study of the Dynamic Tensile Properties of Water-Saturated Sandstone with Different Length-to-Diameter Ratios. Soil Dyn. Earthq. Eng..

[19] Masoumi H., Saydam S., Hagan P.C. (2016). Unified size-effect law for intact rock. Int. J. Geomech..

[20] Zhang Q., Zhu H.H., Zhang L.Y., Ding X.B. (2011). Study of scale effect on intact rock strength using particle flow modeling. Int. J. Rock Mech. Min. Sci..

[21] Yoshinaka R., Osada M., Park H., Sasaki T., Sasaki K. (2008). Practical determination of mechanical design parameters of intact rock considering scale effect. Eng. Geol..

[22] ASTM International (2003). Annual Book of ASTM Standards.

[23] Kahraman S., Alber M. (2006). Estimating unconfined compressive strength and elastic modulus of a fault breccia mixture of weak blocks and strong matrix. Int. J. Rock Mech. Min. Sci..

[24] Hawkins A.B. (1998). Aspects of rock strength. Bull. Eng. Geol. Environ..

[25] Zhu Z.D., Xing F.D., Wang J., Zhang A.J. (2004). Experimental study on size effect on compression strength of brittle rock based on grey theory. Rock Soil Mech..

[26] Yang S.Q., Su C.D., Xu W.Y. (2005). Experimental and theoretical study of size effect of rock material. Eng. Mech..

[27] Özkan İ., Özarslan A., Geniş M., Özşen H. (2009). Assessment of scale effects on uniaxial compressive strength in rock salt. Environ. Eng. Geosci..

[28] Liu X.Y., Li Z.L., Tian W.J., Yu L.X. (2013). Analysis on condition for application of conversion formula in rock uniaxial compressive strength. Yellow River.

[29] Liang C.Y., Li X., Zhang H., Li S.D., He J.M., Ma C.F. (2013). Research on size effect of uniaxial compression properties of granite under medium and low strain rates. Chin. J. Rock Mech. Eng..

[30] Wei J.Z., Liang F.M., Yan S., Li H.B., Yan Z.G. (2018). Experiment on influence of end friction on size effect of sandstone. Coal Mine Saf..

[31] Ping Q., Zhang H., Su H.P. (2018). Study on dynamic compression mechanical properties of limestone with different lengths. Chin. J. Rock Mech. Eng..

[32] Jessu K.V., Spearing A.J.S. (2019). Direct strain evaluation method for laboratory-based pillar performance. J. Rock Mech. Geotech. Eng..

[33] Du K., Su R., Tao M., Yang C.Z., Momeni A., Wang S.F. (2019). Specimen shape and cross-section effects on the mechanical properties of rocks under uniaxial compressive stress. Bull. Eng. Geol. Environ..

[34] Mogi K. (1966). Some precise measurements of fracture strength of rocks under uniform compressive stress. Felsmech. Ingenieurgeol..

[35] Obert L., Duvall W.I. (1967). Rock Mechanics and the Design of Structures in Rock.

[36] Liu B.C., Zhang J.S., Du Q.Z., Tu J.F. (1998). Size effect of rock compressive strength. Chin. J. Rock Mech. Eng..

[37] Yang S.Q., Xu W.Y., Su C.D. (2005). Study on statistical damage constitutive model of rock considering scale effect. Chin. J. Rock Mech. Eng..

[38] Mogi K. (2006). Experimental Rock Mechanics.

[39] Hatheway A.W. (2009). The complete ISRM suggested methods for rock characterization, testing, and monitoring; 1974–2006. Environ. Eng. Geosci..

[40] Hoek E., Brown E.T. (1980). Empirical strength criterion for rock masses. J. Geotech. Eng..

[41] Sujatono S. (2024). Optimum length-to-diameter ratio of floatstone under uniaxial compressive strength test. Geotech. Geol. Eng..

[42] Contreras S., Saldaña M., Toro N., Pérez-Rey I., González M.A., González J. (2021). Scale effect and correlation between uniaxial compressive strength and point load index for limestone and travertine. Appl. Sci..

[43] Hodgson K., Cook N.G.W. The effects of size and stress gradient on the strength of rock. Proceedings of the 2nd ISRM Congress.

[44] Obert L., Windes S.L., Duvall W.I. (1946). Standardized Tests for Determining the Physical Properties of Mine Rocks.

[45] Mogi K. (1962). The influence of the dimensions of specimens on the rock fracture strength of rocks. Bull. Earthq. Res. Inst. Tokyo Univ..

[46] Bieniawski Z.T. (1968). The effect of specimen size on compressive strength of coal. Int. J. Rock Mech. Min. Sci. Geomech. Abstr..

[47] Pratt H.R., Black A.D., Brown W.S., Brace W.F. (1972). The effect of specimen size on the mechanical properties of unjointed diorite. Int. J. Rock Mech. Min. Sci..

[48] Hoskins J.R., Horino F.G. (1969). Influence of Spherical Head Size and Specimen Diameters on the Uniaxial Compressive Strength of Rocks.

[49] Chen J., Yang R., Kang Y. (2020). Influence of the Rock Length-to-Diameter Ratio and Failure Modes on Uniaxial Compression Strength. Geotech. Geol. Eng..

[50] Zhu Q.Z., Min Z.Z., Wang Y.Y., Wang W. (2019). Study on the size effect of silty sandstone samples under conventional triaxial compression. Chin. J. Rock Mech. Eng..

[51] González-Fernández M.A., Estévez-Ventosa X., Alejano L.R., Masoumi H. (2023). Size-Dependent Behaviour of Hard Rock Under Triaxial Loading. Rock Mech. Rock Eng..

[52] Aubertin M., Li L., Simon R. (2000). A multiaxial stress criterion for short- and long-term strength of isotropic rock media. Int. J. Rock Mech. Min. Sci..

[53] Wijk G., Rehbinder G., Lögdström G. (1978). The Relation between the Uniaxial Tensile Strength and the Sample Size for Bohus Granite. Rock Mech..

[54] Coviello A., Lagioia R., Nova R. (2005). On the Measurement of the Tensile Strength of Soft Rocks. Rock Mech. Rock Eng..

[55] Jensen S.S. (2016). Experimental Study of Direct Tensile Strength in Sedimentary Rocks. Master’s Thesis.

[56] Su H.J., Jing H.W., Zhao H.H., Yin Q. (2015). Study on Tensile Strength and Size Effect of Red Sandstone after High Temperature Treatment. Rock Mech. Rock Eng..

[57] Yin Q., Zhao H.H., Deng T.C., Su H.J., Chen Y.S. (2014). Experimental Study on the Influence of Geometry Size on Tensile Strength of Rock. Coal Mine Saf..

[58] Xu Y.F., Zhao F.J., Wang G.J., Zhou S.M. (2012). Size Effect of Brazilian Splitting Strength for Different Rocks. Miner. Eng. Res..

[59] Xu K.L., Liu C.Y., Ni X., Zhu Y., Wan L.P., Deng H.F. (2021). Size Effect on Brazilian Splitting Tensile Strength of Sandstone. J. Yangtze River Sci. Res. Inst..

[60] AlAwad M.N.J. (2022). Modification of the Brazilian Indirect Tensile Strength Formula for Better Estimation of the Tensile Strength of Rocks and Rock-Like Geomaterials. J. King Saud Univ. Eng. Sci..

[61] He W.H., Chen K., Zhang B.Y., Dong K.J. (2018). Improving Measurement Accuracy of Brazilian Tensile Strength of Rock by Digital Image Correlation. Rev. Sci. Instrum..

[62] Wei M.H., Song D.Z., He X.Q., Qiu L.M., Tong Y.J., Lu J.Y. (2025). Acoustic and Electromagnetic Emission Laws of Rocks Affected by Size-Dependent Failure Behavior Under Brazilian Tests. Eng. Fail. Anal..

[63] Song H.H., Zhao Y.X., Wang J.H., Jiang Y.D. (2021). Experimental Investigation of Microstructure-Related Scale Effect on Tensile Failure of Coal. Nat. Resour. Res..

[64] Tavallali A., Vervoort A. (2010). Effect of Layer Orientation on the Failure of Layered Sandstone under Brazilian Test Conditions. Int. J. Rock Mech. Min. Sci..

[65] Basu A., Mishra D.A., Roychowdhury K. (2013). Rock Failure Modes under Uniaxial Compression, Brazilian, and Point Load Tests. Bull. Eng. Geol. Environ..

[66] Wang J., Xie L., Xie H., Ren L., He B., Li C., Yang Z., Gao C. (2016). Effect of Layer Orientation on Acoustic Emission Characteristics of Anisotropic Shale in Brazilian Tests. J. Nat. Gas Sci. Eng..

[67] Vervoort A., Min K.-B., Konietzky H., Cho J.-W., Debecker B., Dinh Q.-D., Frühwirt T., Tavallali A. (2014). Failure of Transversely Isotropic Rock under Brazilian Test Conditions. Int. J. Rock Mech. Min. Sci..

[68] Hong L., Li X.B., Ma C.D., Yin T.B., Ye Z.Y., Liao G.Y. (2008). Study on Size Effect of Rock Dynamic Strength and Strain Rate Sensitivity. Chin. J. Rock Mech. Eng..

[69] Kao S., Zhao G.M., Xu W.S., Cheng X., Dong C.L., Zhang R.F. (2020). Experimental Study of the Association Between Sandstone Size Effect and Strain Rate Effect. J. Mech. Sci. Technol..

[70] Huang B.F., Fu S., Xiao Y. (2021). Uniaxial Compressive Behavior of Granite at High Strain Rates. Rock Mech. Rock Eng..

[71] Zou C.J., Wong L.N.Y. (2016). Size and Geometry Effects on the Mechanical Properties of Carrara Marble Under Dynamic Loadings. Rock Mech. Rock Eng..

[72] Zou C.J., Cheng Y., Li J.C. (2021). Strain Rate and Size Effects on the Brittleness Indexes of Carrara Marble. Int. J. Rock Mech. Min. Sci..

[73] Qi C.Z., Wang M.Y., Bai J.P., Li K. (2014). Mechanism underlying dynamic size effect on rock mass strength. Int. J. Impact Eng..

[74] Qi C.Z., Wang M.Y., Wang Z.F., Li X.Z. (2023). Study on the coupling effect of sample size and strain rate on rock compressive strength. Rock Mech. Rock Eng..

[75] Qi C.Z., Yan F.Y., Zhao F., Li X.Z., Chen H.X. (2022). On the nature of energy-horizon and determination of length scales in dynamic fragmentation of rocks. Int. J. Impact Eng..

[76] Wu Y.Z., Sun Z.Y., Fu Y.K. (2022). Mechanical properties and energy dissipation laws of coal samples with different length-to-diameter ratios under 3D coupled static and dynamic loads. Chin. J. Rock Mech. Eng..

[77] Liu X.D., Liu Z.X. (2024). Effects of Length-to-Diameter Ratio and Strain Rate on Strain Energy Accumulation and Dissipation in Sandstone. Front. Earth Sci..

[78] Koyama T., Jing L. (2007). Effects of model scale and particle size on micro-mechanical properties and failure processes of rocks—A particle mechanics approach. Eng. Anal. Bound. Elem..

[79] Tang C.A., Liu H., Lee P.K.K., Tsui Y., Tham L.G. (2000). Numerical studies of the influence of microstructure on rock failure in uniaxial compression—Part I: Effect of heterogeneity. Int. J. Rock Mech. Min. Sci..

[80] Chen P., Zhou Z.W. (2012). Size effect experiment of rock material with RFPA 2D. J. Liaoning Tech. Univ. Nat. Sci..

[81] Huang Y.H., Yang S.Q., Liu X.R. (2014). Experimental and numerical simulation study on the mechanical properties of rock-like materials. Exp. Mech..

[82] Yang S.Q., Xu W.Y. (2004). Numerical simulation of the size effect on rock material strength under different confining pressures. J. Hohai Univ. Nat. Sci..

[83] Tang W., Zhao X.B., Lei J.Y., Yuan B., Liu H.W. (2016). Numerical Simulation on the Size Effect of Compressive Strength and Deformation Parameters of Rock Materials Under Different Confining Pressures. Geol. J. China Univ..

[84] Peng K., Wu T., Wang Y.M., Luo K., Luo S., Ren J. (2024). Mechanical Behavior and Energy Characteristics of Red Sandstone with Different Seawater Immersion Heights under Biaxial Compression. Phys. Fluids.

[85] Yin T.B., Li X.B., Wang B., Yin Z.Q., Jin J.F. (2011). Mechanical Properties of Sandstones after High Temperature under Dynamic Loading. Chin. J. Geotech. Eng..

[86] Wang Z.H. (2023). Study on Dynamic Mechanical Properties of Red Sandstone Under Freezing and Thawing Cycles. Master’s Thesis.

[87] Xie H.P., Zhu J.B., Zhou T., Zhao J. (2021). Novel Three-Dimensional Rock Dynamic Tests Using the True Triaxial Electromagnetic Hopkinson Bar System. Rock Mech. Rock Eng..

[88] Ma T.X., Chen H., Zhang K., Shen L.X. (2025). The Rheological Intelligent Constitutive Model of Debris Flow: A New Paradigm for Integrating Mechanics Mechanisms with Data-Driven Approaches by Combining Data Mapping and Deep Learning. Expert Syst. Appl..

[89] Ma T.X., Hu X.Q., Liu H.Y., Peng K., Lin Y., Chen Y., Luo K., Xie S.J., Han C.C., Chen M.L. (2025). Elastic Modulus Prediction for High-Temperature Treated Rock Using Multi-Step Hybrid Ensemble Model Combined with Coronavirus Herd Immunity Optimizer. Measurement.

[90] Xie S., Jiang Z., Lin H., Ma T.X. (2024). A New Integrated Intelligent Computing Paradigm for Predicting Joints Shear Strength. Geosci. Front..

[91] Peng K., Zhang Y., Wang Y., Luo S. (2024). Static compression behavior and strength weakening mechanism of dynamically damaged granite after water soaking. Eng. Fail. Anal..

